# Temperature probe placement in very preterm infants during delivery room stabilization: an open-label randomized trial

**DOI:** 10.1038/s41390-024-03115-5

**Published:** 2024-03-05

**Authors:** Pranav R. Jani, Rajesh Maheshwari, Hannah Skelton, Patricia Viola, Sheela Thomas, Lynette Ryder, Mihaela Culcer, Umesh Mishra, Swapnil Shah, Jane Baird, James Elhindi, Ann-Maree Padernia, Traci-Anne Goyen, Daphne D’Cruz, Melissa Luig, Dharmesh Shah

**Affiliations:** 1 Department of Neonatology, Westmead Hospital, Westmead, NSW Australia; 2 The Reproduction and Perinatal Centre, Faculty of Medicine and Health, The University of Sydney, Sydney, NSW Australia; 3 School of Nursing and Midwifery, Western Sydney University, Penrith, NSW Australia; 4 Department of Maternal and Fetal Medicine, Westmead Hospital, Westmead, NSW Australia; 5 Research and Education Network, Westmead Hospital, Westmead, NSW Australia

## Abstract

**Background:**

Variation in practice exists for temperature probe positioning during stabilization of very preterm infants (<32 weeks gestation). We explored the influence of temperature probe sites on thermoregulation.

**Methods:**

An open-label, stratified, balanced, parallel, randomized trial was conducted. Inborn infants were randomly assigned temperature probe to the axilla or to the upper back. The primary outcome was normothermia (local range: 36.8–37.3 °C and World Health Organization (WHO) range: 36.5–37.5 °C) at admission to the neonatal intensive care unit.

**Results:**

Between 1 November 2018 and 4 July 2022, 178 infants were randomly assigned to one of the two sites (*n* = 89 each), 175 included in the final analysis. Normothermia (local range) was achieved for 39/87 infants (44.8%) assigned to the upper back compared to 28/88 infants (31.8%) assigned to the axilla [risk difference:13%; 95% CI −1.3–27.3]. Normothermia (WHO range) was achieved for 78/87 infants (89.7%) assigned to the upper back compared to 70/88 infants (79.6%) assigned to the axilla [risk difference:10.1%; 95% CI −0.5–20.7]. No infant recorded temperatures >38 °C or developed skin injury.

**Conclusions:**

In very preterm infants, upper back site was equally effective as the axilla in maintaining normothermia, with no increase in adverse events.

**Clinical trial registration:**

The study was registered with the Australian New Zealand Clinical Trials Registry (ACTRN12620000293965).

**Impact:**

- Substantial variation in practice exists for the site of securing a temperature probe during delivery room stabilization of very preterm infants and the influence of temperature probe site on thermoregulation remains unknown.
- In this study, upper back site was equally effective as the axilla in maintaining normothermia, with no increase in adverse events.
- Clinicians could adopt upper back site for maintaining normothermia.
- This study may contribute data to future international participant data prospective meta analysis of randomized controlled trials worldwide on temperature probe positioning in very preterm infants, increasing translation of research findings to optimize thermoregulation and clinical outcomes.

## Introduction

The World Health Organization (WHO) defines normothermia as a body temperature between 36.5 °C and 37.5 °C.^1^ For preterm infants born before 37 completed weeks gestational age (GA), hypothermia (body temperature <36.5 °C) soon after birth is a common problem.^2–4^ This issue exists despite the use of evidence-based interventions for the maintenance of normothermia during delivery room (DR) stabilization. These interventions, which are often used in combination, include the use of plastic bags, polyethylene wraps, or caps, and heated humidified gases for respiratory support.^5–8^ Hypothermia is associated with short- and long-term complications. Laptook et al. demonstrated an inverse association between body temperature and in-hospital mortality for preterm infants who were born <34 weeks GA.^2^ Similarly, investigators from the Canadian Neonatal Network reported increased odds of death, or severe neurodevelopmental impairment in preterm infants < 29 weeks GA who were hypothermic upon admission to the neonatal intensive care unit (NICU).^4^ Tay et al. reported hypothermia on admission to the NICU to be an independent predictor for mortality and necrotizing enterocolitis in a large cohort of extremely preterm infants from New South Wales and the Australian Capital Territory in Australia.^3^

Variation in practice exists for the site of securing a temperature probe during DR stabilization.^9^ Bensouda et al. found comparable body temperatures at admission to the NICU when three different sites of temperature probe positioning (left lower back, left upper thorax, and left axilla) were compared in preterm infants ≥28 weeks GA.^10^ In our unit, despite an improvement in normothermia after introducing a thermoregulation bundle for very preterm infants (<32 weeks GA) during DR stabilization, some infants continued to develop hypothermia.^11^ The current study was designed to test the hypothesis that application of a temperature probe to the left upper back during DR stabilization will achieve a higher proportion of normothermia at the time of NICU admission than application in the left axilla in very preterm infants. A systemic review reported on the need for further research to address this question.^12^

The current study was therefore undertaken to compare the effect of securing a temperature probe (TEMPP) at the left upper back versus the left axilla on normothermia at admission to the NICU in preterm infants <32 weeks GA during DR stabilization. Upper back is a standard site for securing a temperature probe,^9^ is readily accessible, is not exposed to the ambient environment and the chances of accidental dislodgement of the temperature probe during the infant’s resuscitation is potentially lower compared to the axilla, therefore we chose the left upper back as the intervention site.

## Methods

### Study design

This was an open-label, single-center, stratified (groups were based on GA), balanced, parallel-group randomized trial. The study was conducted at the Department of Neonatology, within the Women’s and Newborn Health division at Westmead Hospital in Sydney, Australia. This tertiary referral hospital delivers healthcare to more than a million people living in the Local Health District, including perinatal services to about 6000 women each year. This study commenced after obtaining approval from The Western Sydney Local Health District’s Human Research Ethics Committee (HREC/18/WMEAD/192). This study was registered with the Australian New Zealand Clinical Trials Registry (ACTRN12620000293965). The CONSORT 2010 checklist is included in the supplementary file (Supplementary File 1).

### Participants

Inborn infants born between 23^+0/7^ and 31^+6/7^ weeks were eligible to participate in the study. Infants were excluded if they had a severe congenital malformation, were born before the arrival of the NICU team, or if they had a congenital cutaneous malformation at the site of securing the temperature probe. Also, we excluded infants (post-randomization) who had birth asphyxia requiring administration of cardiac compression and adrenaline to ensure a homogenous cohort of infants. These infants were excluded post-randomization for two reasons: i. the maintenance of normothermia in the DR may have been affected by frequent unwrapping of polyethylene wrap for cardiovascular and respiratory assessment and/or for procedures such as umbilical venous catheterization and ii. some infants at this gestation may have died in the DR. No changes were made to the eligibility criteria after the commencement of the study. A research team member introduced the study to the parent in the antenatal ward, or in the birthing unit. Parents provided written informed consent.

### Randomization and masking

Infants were randomly assigned in a 1:1 ratio to receive the intervention at the upper back or at the axilla, using computer-generated random permuted blocks with varying block sizes. The randomization sequence was generated by an independent researcher. Randomization and assignment of the intervention were performed by the attending medical team in the DR, by opening a sequentially numbered, sealed, opaque envelop just before the birth of the infant. Randomization was stratified according to the GA at birth (Group 1: 23^+0/7^– 27^+6/7^ or Group 2: 28^+0/7^–31^+6/7^ weeks). Twin or triplet births were randomized as individual participants. The attending medical team members were not blinded to the intervention. The assigned intervention was documented in the medical records. The data collector, and the statistician were not blinded to the intervention. The data collector had an additional role as medical record custodian for the department to ensure accuracy of the collated clinical records.

### Intervention

**Fig. 1:**
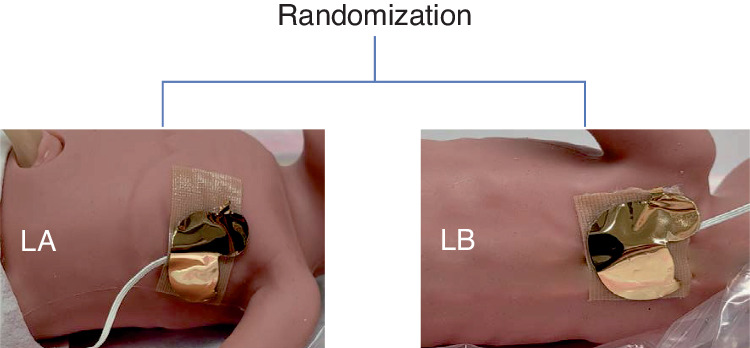
Sites of temperature probe positioning. LA left axilla, LB left upper back.

Infants were randomly assigned to the left upper back (intervention site), or to the left axilla (usual care) for securing the temperature probe. Immediately after the birth the infant was placed supine on the resuscitaire, the nursing team member secured the temperature probe to the allocated site using a soft silicone tape, and a reflector cover (Fig. 1). Immediately after securing the temperature probe, the infant was wrapped in a polyethylene warp for maintaining normothermia. Apart from the interventions, all infants were resuscitated as per the Australian Resuscitation Council’s resuscitation guideline.^13^ This included attempting delayed cord clamping for at least 60 seconds (in the absence of any contraindication), placing the infant without drying in a polyethylene sheet, and using warmed, humidified resuscitation gases. Our unit’s thermoregulation practice for infants born <32 weeks has been described in detail elsewhere.^11^

Measures for maintenance of normothermia for all infants were:

1. using a high-risk infant resuscitaire, and transport shuttle. The infant resuscitaire was connected to the transport shuttle which provided uninterrupted auxillary power to the resuscitaire during transportation of infants. The resuscitaire was equipped to provide advanced resuscitation where required.
2. Using the servo-controlled heat mode (also called baby mode). In this mode, the radiant warmer controlled heat output to maintain a desired set temperature of 37 °C.
3. Wrapping infants in a polyethylene sheet in the DR and ensuring infants were wrapped during transport to the NICU.
4. Using polyethylene-lined bonnet to cover the head and reduce evaporative heat loss.

All infants were positioned supine during DR stabilization and during transport to the NICU. We were unable to adjust the ambient temperature of the DR. After DR stabilization, the open resuscitaire was connected to the transport shuttle, and ongoing heat was delivered during transport to the NICU. A continuous display of the infant’s skin temperature and the set temperature was visible on the resuscitaire’s screen. The resuscitaire did not have an inbuilt capacity to store this information for data download. Our NICU is not co-located with the DR (birthing unit or operating theater), and transportation of an infant after birth from the DR to the NICU takes 10–15 min.

### Outcomes

The primary outcome was the proportion of normothermia in infants at the time of admission to the NICU. This was defined as our local clinical temperature range of 36.8–37.3 °C. In our local thermoregulation guideline, we chose this temperature range to achieve a target admission body temperature near 37 °C. To ensure generalizability of the results, we also used the WHO temperature range of 36.5–37.5 °C for comparison. The bedside nurse recorded the body temperature using a locally available digital thermometer (accuracy of ±0.1 °C) at the infant’s axilla immediately after admission to the NICU, and before transferring the infant to the humidicrib.

The predefined secondary outcomes were death before hospital discharge, major intraventricular hemorrhage defined as grade III or IV based on Papile’s classification, early onset culture-proven sepsis (within the first 48 h after birth), late onset culture-proven sepsis (beyond 48 h of age), ≥ stage II necrotizing enterocolitis based on modified Bell’s classification, cystic periventricular leukomalacia on medical imaging, retinopathy of prematurity needing surgery, and neonatal chronic lung disease defined as continued requirement of either supplemental oxygen, and/or assisted ventilation at 36 weeks of postmenstrual age (PMA).^14–16^ These details were collected from a clinical database which collates maternal, perinatal, and neonatal clinical data verified by designated audit officers following standardized definitions for clinical outcomes.

The ancillary outcomes were fractional oxygen requirement at admission and the maximum fractional oxygen requirement in the first 24 h after birth, first blood sugar level at admission to the NICU, and the need for inotropic support in the first 72 h. Hyperthermia at admission to the NICU (body temperature >38 °C), and any skin injury from the temperature probe during the intervention period were considered adverse events related to the intervention.

### Statistical analysis

A previous audit at the study site showed that 45% of infants born <32 weeks GA were normothermic upon admission to the NICU when following current internationally accepted DR practices for maintaining normothermia. A sample size of 89 infants in each group will provide 80% power, and a two-tailed alpha of 0.05 to detect an improvement to 75% in normothermia. Analysis of the primary outcome was performed following the principles of intention-to-treat analysis. Criteria for excluding infants post-randomization were pre-specified. The analysis of the primary outcome was adjusted for randomization strata based on GA. We documented resuscitaire equipment failure, which was not considered to be a protocol violation. We compared the primary outcome, and the secondary outcomes using an exact test for the difference of proportions between two independent samples. Proportions, counts, absolute risk reduction, and a 95% confidence interval were reported. The number needed to treat (NNT) was additionally reported for the primary outcomes. As a sensitivity analysis, generalized estimating equations equipped with a logit link function modeled the likelihood of normothermia by treatment. We included adjustments for the fixed effect of environmental temperature at birth, and for the random effect of multiple infants within the same pregnancy as these were potential sources of error. A term for the interaction between intervention, and GA group was also added. Significance of the interaction term would support observed differences in subgroup analyses. Statistical analyses were completed in Stata SE Version 14.2 (StataCorp, College Station, TX). All hypotheses were conducted at a significance level of 0.05 with a two-sided alternative. An external data and safety monitoring board was not formed as the study intervention was a low-risk intervention. Two study investigators monitored enrolled patients for the development of any adverse events.

## Results

**Fig. 2:**
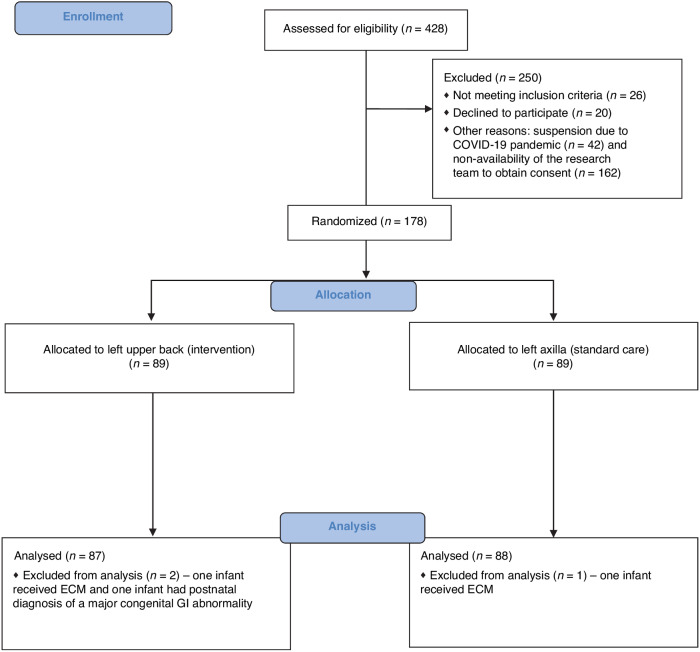
CONSORT flow diagram: Assessment for eligibility, randomization, and analysis. COVID-19 Coronavirus disease, ECM external cardiac massage.

Recruitment commenced on 1 November 2018 and ceased on 4 July 4 2022. We randomly assigned 178 infants equally to receive the temperature probe either at the left upper back or at the left axilla site. Three infants were excluded from the analyses post-randomization; the reasons for their exclusion are shown in the CONSORT flow diagram (Fig. 2). A total of 175 infants (87 infants in the left upper back site and 88 infants in the left axilla site) were included in the primary intention-to-treat analysis, and assessed for the primary outcome. The trial ended as the total sample size was recruited.

The baseline demographic and clinical characteristics of the infants, and their mothers were similar between the two intervention sites (Table 1). The baseline demographic and clinical characteristics of the infants, and their mothers for the two subgroups based on GA were also similar (Table 1). The cohort’s median age at birth was 28 weeks (interquartile range 27–30), and the median birth weight was 1171 g (interquartile range 915–1411). The mean ambient temperature of the birthing unit was higher compared to that of the operation theater by 2.2 °C [22.8 °C (standard deviation 0.9) compared to 20.6 °C (standard deviation 1.2), *p* < 0.01].

**Table 1:** Maternal and infant demographics, baseline clinical characteristics for the cohort, and for the subgroups based on GA.

	Whole cohort		Group 1 (infants ≤27^+6/7^ weeks)		Group 2 (infants ≥28^+0/7^ weeks)	
Characteristic	Left upper back site (*n* = 87)	Left axilla site (*n* = 88)	Left upper back site (*n* = 31)	Left axilla site (*n* = 31)	Left upper back site (*n* = 56)	Left axilla site (*n* = 57)
GA at birth, median (IQR)	29 (27–30)	28 (27–30)	26 (25–27)	26 (26–27)	30 (29–31)	30 (28–31)
BW, g, median (IQR)	1240 (850–1485)	1125 (958–1350)	844 (739–1000)	967 (750–1040)	1410 (1242–1575)	1250 (1112–1450)
Clinically suspected, and/or histopathological chorioamnionitis, *n* (%)	29 (33.3)	25 (28.4)	13 (41.9)	13 (41.9)	16 (28.6)	12 (21)
Received any antenatal steroids (incomplete, complete, >7 days)	86 (98.9)	87 (98.9)	30 (96.8)	31 (100)	56 (100)	56 (98.2)
Caesarean section birth, *n* (%)	65 (74.7)	66 (75)	23 (74.2)	21 (67.7)	42 (75)	45 (78.9)
Ambient temperature of the delivery^a^ room, median (IQR)	21 (20–22.9)	21 (20–22)	21.2 (20–23)	21 (20–22.9)	21 (20–22.5)	21 (20–21.9)
Small for GA, *n* (%)	7 (8)	10 (11.4)	1 (3.2)	1 (3.2)	6 (10.7)	9 (15.8)
Multiple births, *n* (%)	30 (34.5)	22 (25)	10 (32.3)	8 (25.8)	20 (35.7)	14 (24.6)
Male infants, *n* (%)	51 (58.6)	51 (58)	18 (58)	18 (58)	33 (58.9)	33 (57.9)
Time to NICU admission, minutes, median (IQR)	33 (26–40)	30.5 (27–40)	32 (27–40)	34 (29–45)	33 (23.5–40)	30 (26–38)
Infants with an Apgar score <7 at 5 min, *n* (%)	18 (20.7)	20 (23)	11 (35.5)	14 (45.2)	7 (12.5)	6 (10.5)
Received delayed cord clamping, *n* (%)	54 (62)	54 (61.4)	16 (51.6)	19 (61.3)	38 (67.9)	35 (61.4)
Transported from DR on CPAP, *n* (%)	55 (63.2)	53 (60.2)	9 (29)	10 (32.3)	46 (82.1)	43 (75.4)

Data are *n* (%) or median (IQR).

*GA* gestational age, *BW* birth weight, *IQR* interquartile range, *NICU* neonatal intensive care unit, *DR* delivery room, *CPAP* continuous positive airway pressure.

^a^Ambient temperature of the DR was available for 159 infants.

Complete data were available for analyzing the primary outcome for all 175 infants. Normothermia at admission to the NICU when following the local temperature range (36.8 °C–37.3 °C) was achieved in 39 out of 87 (44.8%) infants who were assigned to the left upper back site compared to 28 out of 88 (31.8%) who were assigned to the left axilla site (absolute risk difference: 13%, 95% CI −1.3–27.3, relative risk 1.12, *p* = 0.06). In the subgroup analyses, the effect of the intervention on the proportion of normothermia was greater for infants ≤ 27^+6/7^ weeks (absolute risk difference: 32.3%, 95% CI 9.4–55.1; NNT: 3) compared to infants ≥28^+0/7^ weeks (Table 2). Normothermia at admission to the NICU when following the WHO temperature range (36.5 °C–37.5 °C) was achieved in 78 out of 87 (89.7%) infants who were assigned to the left upper back site compared to 70 out of 88 (79.6%) who were assigned to the left axilla site (absolute risk difference: 10.1%, 95% CI −0.5–20.7). In the subgroup analyses, the effect of the intervention on the proportion of normothermia was higher but not significant for both subgroups (Table 2). For the primary outcome, a sensitivity analysis with adjustment for ambient temperature at birth, and clustering of multiple births was performed. We found no difference in the proportion of normothermia at admission using the WHO temperature range including for the subgroups. A difference was observed when we used the local temperature range for infants in Group 1 but not for Group 2. No independent association was observed between the ambient temperature and normothermia (using either range).

**Table 2:** Primary outcome and subgroup (based on GA at birth) analyses.

	Whole cohort					Group 1 (infants ≤ 27^+6/7^ weeks)					Group 2 (infants ≥ 28^+0/7^ weeks and < 32^+0/7^ weeks)				
Primary outcome	Left upper back site (*n* = 87)	Left axilla site (*n* = 88)	Difference (%), 95% CI	*p* value	NNT	Left upper back site (*n* = 31)	Left axilla site (*n* = 31)	Difference (%), 95% CI	*p* value	NNT	Left upper back site (*n* = 56)	Left axilla site (*n* = 57)	Difference (%), 95% CI	*p* value	NNT
Normothermia (WHO range; 36.5–37.5 ^o^C)	78 (89.7)	70 (79.6)	10.1 (−0.5–20.7)	0.09	–	27 (87.1)	22 (70.9)	16.1 (−3.7–36)	0.21	–	51 (91)	48 (84.2)	5.4 (−6.5–17.2)	0.39	–
Normothermia (local range; 36.8–37.3 ^o^C)	39 (44.8)	28 (31.8)	13 (−1.3–27.3)	0.09	–	17 (54.8)	7 (22.6)	32.3 (9.4–55.1)	0.02	3	22 (39.3)	21 (36.8)	1.8 (−16.2–19.8)	0.85	–

Data are mean (standard deviation) or *n* (%). difference = absolute risk reduction.

*CI* confidence interval, *NA* not available, *NNT* number needed to treat (rounded to the nearest whole number only where statistical significance was achieved), *WHO* World Health Organization.

The incidences of pre-specified secondary outcomes were similar for infants assigned to the two interventions including for each of the two GA-based subgroups (Table 3).

**Table 3:** Secondary outcomes.

	Whole cohort				Group 1 (infants ≤ 27^+6/7^ weeks)				Group 2 (infants ≥ 28^+0/7^ weeks)			
Secondary outcomes	Left upper back site (*n* = 87)	Left axilla site (*n* = 88)	*p* value	Difference (%) (95% CI)	Left upper back site (*n* = 31)	Left axilla site (*n* = 31)	*P* value	Difference (%) (95% CI)	Left upper back site (*n* = 56)	Left axilla site (*n* = 57)	*P* value	Difference (95% CI)
Death before Hospital discharge, *n* (%)	1 (1.2)	4 (4.5)	0.37	−3.4 (−8.3–1.5)	1 (3.2)	3 (9.7)	0.61	−6.5 (−18.6–5.7)	0	1 (1.8)	1.00	−1.8 (−5.2–1.7)
Major IVH (≥grade III), *n* (%)	2 (2.3)	4 (4.5)	1.00	−2.3 (−7.6–3.1)	1 (3.2)	4 (12.9)	0.35	−9.7 (−23–3.7)	1 (1.8)	0	1.00	1.8 (−1.7–5.3)
Early onset sepsis (≤48 h of age), *n* (%)	1 (1.2)	0	1.00	1.2 (−1–3.4)	1 (3.2)	0	1.00	3.2 (−3–9.5)	0	0	NA	NA
Late onset sepsis (>48 h of age), *n* (%)	8 (9.2)	7 (8)	0.79	1.2 (−7–13.6)	6 (19.4)	3 (9.7)	0.47	9.6 (−7.7–27)	2 (3.6)	4 (7)	0.68	−3.5 (−11.7–4.8)
NEC ≥stage II, *n* (%)	1 (1.2)	4 (4.5)	0.37	−3.4 (−8.3–1.5)	1 (3.2)	3 (9.7)	0.61	−6.5 (−18.6–5.7)	0	1 (1.8)	1.00	−1.8 (−5.2–1.7)
PVL, *n* (%)	1 (1.2)	4 (4.5)	0.37	−3.4 (−8.3–1.5)	0	3 (9.7)	0.24	−9.7 (−20 – −0.7)	1 (1.8)	1 (1.8)	1.00	0
ROP requiring surgery, *n* (%)	2 (2.5)	3 (3.8)	0.68	−1.3 (−6–3.8)	2 (6.5)	1 (3.5)	1.00	3 (−7.4–13.9)	0	2 (3.5)	0.60	−3.5 (−8.3–1.3)
CLD, *n* (%)	27 (31)	32 (36.4)	0.52	−5.4 (−19.3–8.7)	19 (61.3)	18 (58)	1.00	3.3 (−21.2–27.6)	8 (14.3)	14 (24.6)	0.24	−10.3 (−24.7–4.2)

Data are *n* (%).

*IVH* intraventricular hemorrhage, *NEC* necrotizing enterocolitis, *PVL* periventricular leukomalacia, *ROP* retinopathy of prematurity, *CLD* chronic lung disease, *CI* confidence interval.

**Fig. 3:**
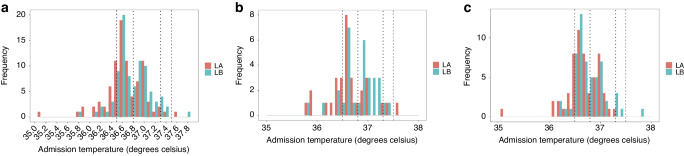
The distribution of mean admission body temperature based on the intervention. **a** For the whole cohort. **b** For Group 1 (infants ≤27^+6/7^ weeks). **c** For Group 2 (infants ≥28^+0/7^ weeks and <32^+0/7^ weeks). LA left axilla, LB left upper back. Broken dash line = 36.5–37.5 °C, dotted line = 36.8–37.3 °C.

Ancillary outcomes: For the whole cohort, the mean body temperature which was measured at admission to the NICU differed significantly for infants assigned to the left upper back compared to the left axilla site (*p* = 0.02, Table 4 and Fig. 3). For both GA based subgroups, although the mean body temperature was higher for infants assigned to the left upper back compared to left axilla site for both subgroups, this difference was not significant. (Table 4 and Fig. 3). When we used WHO’s range for normothermia, hypothermia (body temperature <36.5 °C) was observed in eight out of 87 infants (9.2%) for the upper back compared to 17 out of 88 infants (19.3%) for the axilla. This difference, including for the subgroups, was not significant (Group 1: upper back four out of 31 (12.9%) compared to axilla eight out of 31 (25.8%); Group 2: upper back four out of 56 (7.1%) compared to nine out of 57 (15.8%). The proportion of infants who recorded a body temperature of >37.5 °C was low (1%), and similar for both sites. No infant had local skin injury from temperature probe application, nor did they record body temperature >38 °C at admission to the NICU. There was no difference in the proportion of normothermia in infants based on whether they received delayed cord clamping or not [93/108 (86.1%) versus 55/67 (82%), *p* = 0.52]. The incidence of the other post-hoc ancillary outcomes were similar for infants in both arms of the study. They included: fraction of inspired oxygen at and within the first 24 h of admission, first blood sugar level at admission, and need for inotrope in the first 72 h of admission (Table 4).

**Table 4:** Ancillary outcomes analyses.

	Whole cohort				Group 1 (infants ≤27^+6/7^ weeks)				Group 2 (infants ≥28^+0/7^ weeks)			
Ancillary outcomes^a^	Left upper back site (*n* = 87)	Left axilla site (*n* = 88)	*p* value	Difference (%) (95% CI)	Left upper back site (*n* = 31)	Left axilla site (*n* = 31)	*p* value	Difference (%) (95% CI)	Left upper back site (*n* = 56)	Left axilla site (*n* = 57)	*p* value	Difference (%) (95% CI)
Unadjusted admission temperature in °C, mean (SD)	36.88 (0.3)	36.7 (0.4)	0.02	0.1 (0.02–0.2)	36.8 (0.4)	36.6 (0.4)	0.08	0.2 (−0.02–0.4)	36.8 (0.3)	36.7 (0.3)	0.13	0.09 (−0.03–0.2)
Hypothermia (<36.5 °C)	8 (9.2)	17 (19.3)	0.08	10.3	4 (12.9)	8 (25.8)	0.34	12.9	4 (7.1)	9 (15.8)	0.24	8.9
Hypothermia (<36.8 °C)	45 (51.7)	57 (65.5)	0.09	13.8	13 (41.9)	22 (71)	0.04	29.1	32 (57.1)	35 (62.5)	0.07	5.4
FiO_2_ at admission, median (IQR)	23 (21–30)	25 (21–30)	0.87	1.1 (−2.5–4.7)	25 (21–30)	24 (21–30)	0.66	−1.9 (−8.0–4.3)	23 (21–30)	25 (21–30)	0.62	2.7 (−1.9–7.3)
Maximum FiO_2_ in the first 24 h, median (IQR)	28 (22–40)	29 (21–40)	0.99	−1.8 (−7.7–4.1)	30 (26–45)	20 (25–40)	0.41	−5.8 (−16.2–4.6)	25 (21–33)	26 (21–35)	0.52	0.4 (−6.7–7.5)
Blood sugar level in mmols/L at admission, median (IQR)	2.7 (1.9–3.9)	2.9 (2.1–3.8)	0.55	0.03 (−0.4–0.5)	3.2 (2.1–4.5)	2.7 (2.2–3.6)	0.25	−0.6 (−1.4–0.3)	2.4 (1.8–3.6)	3 (2–3.8)	0.14	0.3 (−0.1–0.8)
Inotropes in the first 72 h, *n* (%)	5 (5.7)	5 (5.7)	1.0	0.1 (−6.8–6.9)	3 (9.7)	3 (9.7)	1.0	0	2 (3.6)	2 (3.5)	1.0	0.1 (−6.8–6.9)

Difference = absolute risk reduction. Data are *n* (%) or median (IQR).

*IQR* interquartile range, *FiO*_*2*_ fraction of inspired oxygen, *CI* confidence interval.

^a^These outcomes were added post-hoc.

## Discussion

During DR stabilization of preterm infants <32 weeks gestation, compared to the standard site (left axilla), the intervention site (left upper back) had a higher proportion of normothermia (both WHO and local temperature ranges), although this difference was not statistically significant. This effect was consistent across the GA-based subgroups, except for infants ≤27^+6/7^ weeks, in whom the proportion of normothermia was higher when we used a local temperature range for the intervention site compared with standard care site. The rates of pre-specified secondary outcomes were similar between the two groups. A higher mean body temperature at admission to the NICU, and no adverse events such as body temperature ≥38 °C or local skin injury from the temperature probe.

Many interventions are listed for the maintenance of normothermia in preterm infants.^17^ This evidence is, at best, of a moderate quality, and in clinical practice, these interventions are used collectively for maintaining normothermia.^9^ But despite advances in the technology, and availability of equipment for thermoregulation, hypothermia in preterm infants still exists. Need for further research on exploring an ideal site for TEMPP has been highlighted.^12^ There is variation in practice for the site of securing a temperature probe.^9^ We chose the left upper back site for the intervention for the following reasons: i) it is a standard site for TEMPP, ii) easy access for securing the probe during DR stabilization, iii) flat skin surface, and is not exposed to the ambient environment compared to exposed and uneven skin surfaces (from underlying ribs) at the standard site, and iv) potentially a lower chance of accidental dislodgement of the probe during the infant’s resuscitation.^9^ Therefore, the current study was designed to test the hypothesis that application of a temperature probe to the left upper back during DR stabilization will achieve a higher proportion of normothermia at the time of NICU admission than application in the left axilla in very preterm infants. The current study showed a clinically important effect on maintaining normothermia when using the intervention site compared to the standard site for TEMPP. The results of the subgroup analyses suggested a greater benefit of the intervention to infants ≤27^+6/7^ weeks GA, with a NNT of three (for the local temperature range) for one infant to benefit. The reasons for this observation could be better contact between the temperature probe sensor and the flat skin surface at the intervention site, or less exposure of the intervention site to an environmental draught of cold air. This in turn maintained steady perfusion to the site. We observed a bimodal empirical distribution for the body temperature at admission to the NICU. We did not find the variable responsible for this observation.

Globally, of the 13.4 million preterm infants (<37 weeks gestation), ~2 million were born <32 weeks gestation, and 0.6 million were born <28 weeks gestation.^18^ Without immediate high-quality care, most infants <28 weeks will not survive. Servo-control heating mode was used for maintaining normothermia during transport of infants ≤27^+6/7^ weeks by >40% NICUs from low and lower-middle-income, upper-middle-income, and high-income countries.^9^ In the current trial, the intervention was implemented using servo-control heating mode for all infants. Also, a higher proportion of infants assigned to the upper back group maintained normothermia (for the WHO and local range) compared to axilla, but this difference was not significant. This suggests that the present study intervention can be implemented for maintaining normothermia during DR stabilization at hospitals delivering preterm infants and using a servo-control heating mode across all geographic, and income status-based regions.

TEMPP is the first study comparing the effect of securing the temperature probe at the upper back site on thermoregulation in very preterm infants including those before 28 weeks gestation. We achieved very high adherence to the protocol (>99%). While the result of this single-center study is encouraging and applicable to some NICUs, it cannot be generalized to all NICUs. This includes NICUs using the resuscitaire in a manual heating mode during DR stabilization and for transporting infants from the DR to the NICU, those using additional devices to provide warmth such as a heating mattress, and those providing active care to infants <23^+0/7^ weeks gestation. Further prospective research with the above practices or patients needs to be undertaken before changing practice. Both the sites for securing the temperature probe are standard care interventions and carry a low risk. For such comparative effectiveness trials, there is emerging literature regarding the acceptance of a waiver of consent by the parents and the researchers and its role in ensuring a wider representation of the population studied, and the generalizability of the result.^19^ Similar low-risk comparative effectiveness trials in the future may benefit from “waiver of consent or deferred consent” approach if this is not prohibited by state or national law.

We acknowledge several limitations of the current single center trial. Blinding the clinical team in the DR to the assigned intervention was impossible. We also did not blind the data collector or the statistician, to increase rigor in interpreting the analysis, and minimizing errors. We did not collect information on first temperature nor the time to reach normothermia in the DR as this could have distracted the resuscitation team from infant stabilization. However, for infants in the two intervention groups, we compared the time to admission to the NICU as a surrogate marker for reaching normothermia in DR, and they were similar. Also, there were no differences in measures for maintaining normothermia in the DR for all infants. A third of the eligible infants could not be enrolled as a researcher was not available afterhours for obtaining consent, or the delivery occurred rapidly. The study was paused during the pandemic. Three infants met the pre-specified exclusion criteria, hence only 175 infants were included in the final analysis. The sample size calculation was performed using a baseline rate of normothermia and a 30% improvement in the rates of normothermia from clinical practice improvement.^11^ It is possible that the Hawthorne effect and local clinical practice improvement activities may have contributed to an increase in proportion of normothermia in the control group. For these reasons, our results did not show such an outcome despite the small but appropriately powered sample size. Finally, the sample size of infants ≤27^+6/7^ weeks gestation was small, this may have led to wide confidence intervals. This reflects the incidence of birth at this gestation in the local population.

In conclusion, during DR stabilization of preterm infants <32 weeks gestation, securing a temperature probe at the left upper back compared with the left axilla was equally effective in maintaining normothermia with no significant increase in adverse events. Clinicians attending birth of infants <32 weeks, and using servo control heat mode could adopt upper back site for maintaining normothermia. Ultimately, our study may contribute data to future international participant data prospective meta analysis of randomized controlled trials worldwide on temperature probe positioning in very preterm infants, increasing translation of research findings to optimize thermoregulation and clinical outcomes.^20^

### Supplementary Information

Supplementary file 1

## Data Availability

All data generated or analyzed during this study are included in this published article.

## References

[1] World Health Organization. (1993). Thermal control of the newborn: a practical guide.

[2] Laptook AR (2018). Admission Temperature and Associated Mortality and Morbidity among Moderately and Extremely Preterm Infants. J. Pediatr..

[3] Tay VY (2019). Admission temperature and hospital outcomes in extremely preterm infants. J. Paediatr. Child Health.

[4] Ting JY, Synnes AR, Lee SK, Shah PS, Canadian Neonatal Netwrok and Canadian Neonatal Follow-Up Network. (2019). Association of admission temperature and death or adverse neurodevelopmental outcomes in extremely low-gestational age neonates. J. Perinatol..

[5] Leadford AE (2013). Plastic bags for prevention of hypothermia in preterm and low birth weight infants. Pediatrics.

[6] Vohra S, Roberts RS, Zhang B, Janes M, Schmidt B (2004). Heat Loss Prevention (HeLP) in the delivery room: A randomized controlled trial of polyethylene occlusive skin wrapping in very preterm infants. J. Pediatr..

[7] Trevisanuto D (2010). Heat loss prevention in very preterm infants in delivery rooms: a prospective, randomized, controlled trial of polyethylene caps. J. Pediatr..

[8] te Pas AB, Lopriore E, Dito I, Morley CJ, Walther FJ (2010). Humidified and heated air during stabilization at birth improves temperature in preterm infants. Pediatrics.

[9] Jani P (2023). Thermoregulation and golden hour practices in extremely preterm infants: an international survey. Pediatr. Res.

[10] Bensouda B (2018). Temperature Probe Placement during Preterm Infant Resuscitation: A Randomised Trial. Neonatology.

[11] Singh TS (2022). Improvement in thermoregulation outcomes following the implementation of a thermoregulation bundle for preterm infants. J. Paediatr. Child Health.

[12] Joseph RA, Derstine S, Killian M, Gephart S (2017). Ideal site for skin temperature probe placement on infants in the NICU. Adv. Neonatal Care.

[13] Liley, H., Mildenhall, L., Thio, M., Gately, C. ANZCOR Guideline 13.8 – The Resuscitation of the Newborn in Special Circumstances Australia: Australian Resuscitation Council; 2021. https://www.resus.org.nz/assets/Uploads/ANZCOR-Guideline-13.8-April-2021.pdf Accessed 25 Sept 2023.

[14] Papile L-A, Burstein J, Burstein R, Koffler H (1978). Incidence and evolution of subependymal and intraventricular hemorrhage: a study of infants with birth weights less than 1,500 gm. J. Pediatr..

[15] Walsh MC, Kliegman RM (1986). Necrotizing enterocolitis: treatment based on staging criteria. Pediatr. Clin. North Am..

[16] Chow, S. S. W., Creighton, P., Chambers, G. M., Lui, K. Report of the Australian and New Zealand Neonatal Network 2020. https://anznn.net/Portals/0/AnnualReports/Report%20of%20the%20Australian%20and%20New%20Zealand%20Neonatal%20Network%202020_amended.pdf. Accessed 25 Sept 2023.

[17] McCall, E. M., Alderdice, F., Halliday, H. L., Vohra, S., Johnston, L. Interventions to prevent hypothermia at birth in preterm and/or low birth weight infants. *Cochrane Database Syst. Rev.* **2018**, CD004210 *(*2018). 10.1002/14651858.CD004210.pub5 PMC6491068 29431872

[18] World Health Organization. (2023). Born too soon: decade of action on preterm birth.

[19] Rich WD, Katheria AC (2017). Waiver of consent in a trial intervention occurring at birth—how do parents feel?. Front Pediatr..

[20] Seidler AL (2019). A guide to prospective meta-analysis. BMJ.

