# Supplementary material for: Temperature probe placement in very preterm infants during delivery room stabilization: an open-label randomized trial
Source: Pediatr Res. 2024 Mar 5;96(1):190–8. doi: 10.1038/s41390-024-03115-5 (PMC11257937; doi:10.1038/s41390-024-03115-5)
Supplement: Supplementary file 1 [file 41390_2024_3115_MOESM1_ESM.pdf]

CONSORT 2010 checklist of information to include when reporting a randomised trial*

Section/Topic	Item No	Checklist item	Reported on page No
**Title and abstract**			
	1a	Identification as a randomised trial in the title	1
	1b	Structured summary of trial design, methods, results, and conclusions (for specific guidance see CONSORT for abstracts)	2
**Introduction**			
Background and objectives	2a	Scientific background and explanation of rationale	4
	2b	Specific objectives or hypotheses	4/5
**Methods**			
Trial design	3a	Description of trial design (such as parallel, factorial) including allocation ratio	6
	3b	Important changes to methods after trial commencement (such as eligibility criteria), with reasons	6
Participants	4a	Eligibility criteria for participants	6
	4b	Settings and locations where the data were collected	6
Interventions	5	The interventions for each group with sufficient details to allow replication, including how and when they were actually administered	7
Outcomes	6a	Completely defined pre-specified primary and secondary outcome measures, including how and when they were assessed	8
	6b	Any changes to trial outcomes after the trial commenced, with reasons	8
Sample size	7a	How sample size was determined	9
	7b	When applicable, explanation of any interim analyses and stopping guidelines	9/10
Randomisation:			
Sequence generation	8a	Method used to generate the random allocation sequence	6
	8b	Type of randomisation; details of any restriction (such as blocking and block size)	6
Allocation concealment mechanism	9	Mechanism used to implement the random allocation sequence (such as sequentially numbered containers), describing any steps taken to conceal the sequence until interventions were assigned	6/7
Implementation	10	Who generated the random allocation sequence, who enrolled participants, and who assigned participants to interventions	6/7
Blinding	11a	If done, who was blinded after assignment to interventions (for example, participants, care providers, those	6/7

		assessing outcomes) and how	
	11b	If relevant, description of the similarity of interventions	
Statistical methods	12a	Statistical methods used to compare groups for primary and secondary outcomes	9
	12b	Methods for additional analyses, such as subgroup analyses and adjusted analyses	9
**Results**			
Participant flow (a diagram is strongly recommended)	13a	For each group, the numbers of participants who were randomly assigned, received intended treatment, and were analysed for the primary outcome	11
	13b	For each group, losses and exclusions after randomisation, together with reasons	11
Recruitment	14a	Dates defining the periods of recruitment and follow-up	11
	14b	Why the trial ended or was stopped	11
Baseline data	15	A table showing baseline demographic and clinical characteristics for each group	11
Numbers analysed	16	For each group, number of participants (denominator) included in each analysis and whether the analysis was by original assigned groups	11
Outcomes and estimation	17a	For each primary and secondary outcome, results for each group, and the estimated effect size and its precision (such as 95% confidence interval)	11/12, table 2
	17b	For binary outcomes, presentation of both absolute and relative effect sizes is recommended	-
Ancillary analyses	18	Results of any other analyses performed, including subgroup analyses and adjusted analyses, distinguishing pre-specified from exploratory	12
Harms	19	All important harms or unintended effects in each group (for specific guidance see CONSORT for harms)	12
**Discussion**			
Limitations	20	Trial limitations, addressing sources of potential bias, imprecision, and, if relevant, multiplicity of analyses	15
Generalisability	21	Generalisability (external validity, applicability) of the trial findings	14/15
Interpretation	22	Interpretation consistent with results, balancing benefits and harms, and considering other relevant evidence	14-16
**Other information**			
Registration	23	Registration number and name of trial registry	10
Protocol	24	Where the full trial protocol can be accessed, if available	Supplementary
Funding	25	Sources of funding and other support (such as supply of drugs), role of funders	None

*We strongly recommend reading this statement in conjunction with the CONSORT 2010 Explanation and Elaboration for important clarifications on all the items. If relevant, we also recommend reading CONSORT extensions for cluster randomised trials, non-inferiority and equivalence trials, non-pharmacological treatments, herbal interventions, and pragmatic trials. Additional extensions are forthcoming: for those and for up to date references relevant to this checklist, see www.consort-statement.org.

Non-Drug/Device Protocol Template

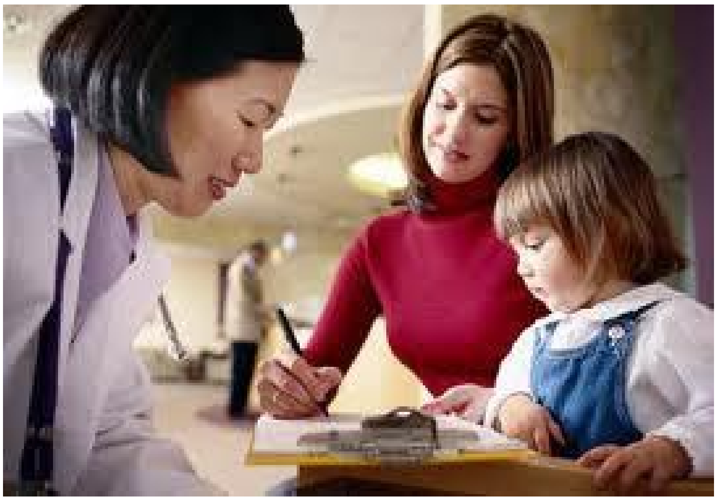

Published Date: August 2013

Review Date: August 2014

NOTES TO USERS

**Who should use this template?**	Anyone conducting clinical research which does **not** involve drugs or devices.
**Why do you need a protocol?**	The protocol is essential for study conduct, review, reporting, and interpretation.
**Why use this template?**	This non-drug template has been modified from the SPRIRIT (**S**tandard **P**rotocol **I**tems: **R**ecommendations for **I**nterventional **T**rials). The Spirit Statement is an international initiative that aims to improve the quality of clinical trial protocols by defining an evidence-based set of items to address in a protocol.<br><br>Reference: Chan et al., (2013) SPIRIT 2013 Explanation and Elaboration: Guidance for protocols of clinical trials. *BMJ 2013; 346:e7586*
**How do I use this template?**	There is a brief explanation under each heading stating the information that should be contained in that section.<br><br>You will need to input your study specific information under each heading and remove explanatory information.<br><br>As this is a template, users are remaindered that not all examples may be applicable to their study. Please contact your institution to discuss specific protocol questions.
**Do I still need to complete the National Ethics Application Form (NEAF)**	Yes – you must finalise your protocol prior to completing the NEAF. The NEAF is a form used by ethics committees to conduct standard review of all projects. While you need to refer to your protocol to answer most questions in the NEAF, it does not replace the need for a detailed protocol.
**Copyright**	This template is licensed under the Creative Commons Attribution NonCommercial-NoDerivs 3.0 Unported License

PROTOCOL

Title: Where should the temperature probe be secured during preterm infant resuscitation? A randomised trial

---

Protocol Number (if applicable):

Version: 3.0

Date: 16/09/2020

Author/s:

Dr Rajesh Maheshwari, Dr Pranav Jani, Dr Dharmesh Shah, Dr Melissa Luig, Ann-Maree Padernia, Jane Baird, Claire Galea

Sponsor/s:

NA

CONFIDENTIAL

This document is confidential and the property of Westmead Hospital. No part of it may be transmitted, reproduced, published, or used without prior written authorization from the institution.

Statement of Compliance

This document is a protocol for a research project. This study will be conducted in compliance with all stipulation of this protocol, the conditions of the ethics committee approval, the NHMRC National Statement on ethical Conduct in Human Research (2007) and the Note for Guidance on Good Clinical Practice (CPMP/ICH-135/95).

Table of Contents

Contents

Table of Contents ... 4

1. **Glossary of Abbreviations & Terms** ... 6
2. **Study Sites** ... 6
   - 2.1 Study Location/s ... 6
3. **Funding and Resources** ... 6
   - 3.1 Source/s of Funding ... 6
4. **Introduction/Background Information** ... 6
   - 4.1 Lay Summary ... 6
   - 4.2 Introduction ... 6
   - 4.3 Background information ... 7
5. **Study Objectives** ... 7
   - 5.1 Research Question ... 7
   - 5.2 Primary Objectives ... 7
   - 5.3 Secondary Objectives ... 7
   - 5.4 Outcome Measures ... 7
6. **Study Design** ... 8
   - 6.1 Study Design Diagram ... 8
   - 6.2 Study Type & Design & Schedule ... 9
   - 6.3 Standard Care and Additional to Standard Care Procedures ... 10
   - 6.4 Randomisation ... 11
   - 6.5 Study methodology ... 11
7. **Study Population** ... 11
   - 7.1 Recruitment Procedure ... 11
   - 7.2 Inclusion Criteria ... 12
   - 7.3 Exclusion Criteria ... 12
   - 7.4 Consent ... 12

8. Participant Safety and Withdrawal 12

8.1 Risk Management and Safety 12

8.2 Adverse Event Reporting 12

8.3 Handling of Withdrawals 12

8.4 Replacements 13

9. Statistical Methods 13

9.1 Sample Size Estimation & Justification 13

9.2 Power Calculations 13

9.3 Statistical Methods To Be Undertaken 13

10. Storage of Blood and Tissue Samples 13

10.1 Details of where samples will be stored, and the type of consent for future use of samples 13

11. Data Security & Handling 13

11.1 Details of where records will be kept & How long will they be stored 13

11.2 Confidentiality and Security 13

11.3 Ancillary data 14

12. Appendix 14

13. References 14

1. Glossary of Abbreviations & Terms

Abbreviation	Description (using lay language)
NICU (Neonatal Intensive Care Unit)	Intensive care unit for babies
ANZNN (Australia and New Zealand Neonatal Network)	Network of neonatal units across Australia and New Zealand

2. Study Sites: Westmead Hospital, Westmead, NSW

2.1 Study Location/s

[List all locations, their address & contact details this study or parts of the study will be conducted]

Site	Address	Contact Person	Phone	Email
Westmead Hospital	PO Box 533, Wentworthville NSW 2 2145	Dr Pranav Jani	8890 6645	Pranav.jani@health.nsw.gov.au

3. Funding and Resources

3.1 Source/s of Funding

No external funding or extra funding is required as the equipment used during the study is part of the routine care of preterm infants.

4. Introduction/Background Information

4.1 Lay Summary

Preterm infants are susceptible to hypothermia (low body temperature) soon after birth due to reduced body fat stores. Abnormal body temperature (high as well as low) upon admission to the neonatal unit has been linked to increased mortality in these infants. Current standard practice at the time of birth involves placing a skin temperature probe in the left armpit that allows the display of body temperature and using a servo mode on the radiant heater whereby the heater automatically regulates the heat output based on the difference between the set temperature (set by the user) and the displayed body temperature. Optimal site for temperature probe placement remains unclear, traditionally left arm pit (axilla) has been used.

In this study, we aim to compare two different sites for the placement of the temperature probe at the time of birth (either left armpit or left upper back) in a randomised manner and compare the admission temperature taken by a thermometer placed in the armpit to see if one of these sites is superior to the other. The primary outcome will be the proportion of babies with normal temperature measured at admission (normal temperature is between 36.8 and 37.3°C). The usual time to admission after birth is about 20-30 minutes. There is no other

variation from the routine care.

4.2 INTRODUCTION

Hypothermia at admission to the NICU remains a common problem in preterm infants. In recent years, research and quality improvement initiatives have provided answers to many questions relating to temperature management in preterm infants at the time of birth (e.g. using plastic cover, use of heated mattress, use of heated humidified gases for resuscitation) however the optimal site on the skin from where to record the body temperature in order to regulate the heater output remains unclear and there are no recommendations made by the resuscitation bodies as well. A recent study compared three different sites for probe placement without finding any significant differences in admission temperatures but that study was conducted in preterm infants born between 28-35 weeks' gestation. For extremely preterm infants (born less than 28 weeks gestation), no information regarding the optimal site for temperature probe placement at the time of birth is available.

This study aims to compare two different sites for temperature probe placement in preterm infants born between 23-31 weeks' gestation. Either left armpit (standard site) or left upper back (experimental site) will be used for an individual infant. The selection of the site will be made in a randomised manner. All other aspects of clinical care will be similar between the two groups as per the current guidelines. The proportion of infants with normal axillary temperature recorded at the time of admission to the neonatal unit will be compared between the 2 groups to see if one site is superior to the other in terms of having more infants with their temperatures in the desired range.

4.3 BACKGROUND INFORMATION

As per the recent Australian and New Zealand Neonatal Network (ANZNN 2015) data, there has been a steady improvement in the survival rates of preterm infants. However, hypothermia (low body temperature) at admission remains a significant problem in preterm infants due to prematurity, low birth weight, poor body fat stores and increased energy demands due to sickness. Abnormal body temperature (outside the range of 36.8-37.3◦C) on admission to the neonatal intensive care unit (usually hypothermia, occasionally hyperthermia or high temperature) has been associated with increased risk of mortality and morbidity. In low birth weight infants, for every 1◦C drop in admission temperature below 36◦C, the likelihood of mortality increases by 28%.[1] In a recent publication[2], the relationship between admission temperature and adverse neonatal outcomes was U-shaped. The lowest rates of adverse outcomes were associated with admission temperatures between 36.5°C and 37.2°C. In a Canadian study[3], the authors compared three different sites of probe placement and did not note any significant differences in admission temperatures. This study however did not include preterm infants <28 weeks gestation. This is the group of preterm infants more likely to be hypothermic on admission to the NICU. The data from our unit for 2016 shows that 45% of preterm infants (<32 weeks gestation) had abnormal temperature at NICU admission.

5. STUDY OBJECTIVES

5.1 RESEARCH QUESTION

Does securing the temperature probe to the left upper back lead to more preterm infants (23-31 weeks'

gestation) having their admission temperature in the target range at the time of admission to the NICU as compared to securing the probe in the left axilla?

5.2 PRIMARY OBJECTIVES

The primary objective is to find out if in a population of preterm infants (23-31 weeks' gestation), securing the temperature probe to the left upper back at the time of birth (intervention) as compared to securing it in the left axilla (comparator) will lead to a greater proportion of infants having their admission temperature in the target range (36.8-37.3°C) at the time of neonatal unit admission (outcome).

5.3 SECONDARY OBJECTIVES

NA

5.4 OUTCOME MEASURES

Primary outcome:
Axillary temperature on admission to the NICU (recorded within a minute of NICU arrival)

Secondary outcomes:
Death
Major intra-ventricular haemorrhage (grade 3 or 4 IVH)
Early onset culture proven sepsis
Late onset culture proven sepsis
Stage II and above necrotising enterocolitis
Cystic peri-ventricular leucomalacia
Retinopathy of prematurity needing surgery
Neonatal chronic lung disease

6. STUDY DESIGN

6.1 STUDY DESIGN DIAGRAM (Randomised controlled study)

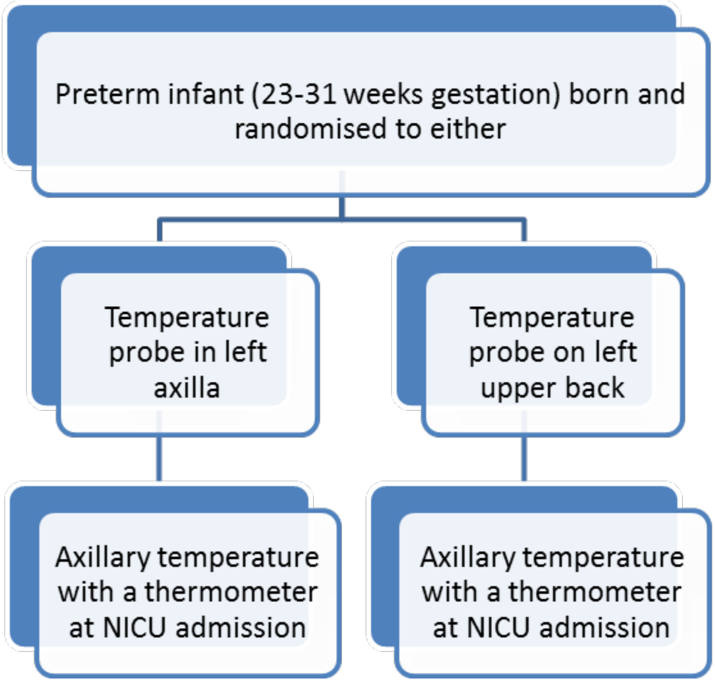

6.2 STUDY TYPE & DESIGN & SCHEDULE

Research project setting: Neonatal Intensive Care Unit, Birthing Unit and Operation Theatre at Westmead Hospital, Sydney, Australia.

Methodological approach: Randomised controlled trial (RCT) to eliminate unknown or unmeasured confounders likely to bias the study results.

Participants: Preterm infants between 23^{+0} weeks and 31^{+6} weeks born at Westmead Hospital. The randomisation will be stratified for two gestation bands (23-27 weeks, 28-31 weeks). This is to ensure groups are balanced for preterm infants of various gestations. Preterm infants from multiple births i.e. twins or triplets will be regarded as independent participants.

Exclusions: Severe congenital malformations, severe asphyxia requiring cardiac compressions and adrenaline administration, birth prior to arrival of the NICU team, congenital cutaneous lesions.

Participant recruitment strategies and timeframes: Study is planned for about 18 months from approval. We plan to conduct education sessions for the medical and nursing team who attend preterm births after the ethics approval.

Approach/es to provision of information to participants and/or consent: This study will be conducted at Westmead Hospital's neonatal intensive care unit. Potential participants will include preterm infants born at Westmead Hospital less than 32 weeks gestation (i.e. 31^{+6} weeks and below). Parents of potentially eligible study participants will be approached upon admission to the hospital for preterm birth. One of the study investigators will approach the family for an informed consent prior to the birth of the baby. For non-English speaking families, interpreter service will be used.

Data collection: The data on admission temperature will be collected from the medical records. The information about the secondary outcomes will be collected from the neonatal unit database as this information is routine collected for benchmarking.

Date storage: The data will be stored in a WSLHD computer which will be password protected. The password will only be known to the members of the research team. The data file will be transferred among the research team members via secure email (only with the health email address). After the required period of storage (7 years), this data will be deleted.

Follow-up: No long-term follow-up is planned as part of this study. The primary outcome related information is available at the time of NICU admission (20-30 minutes of age). The secondary outcome related information is available by the time of discharge from the unit.

6.3 STANDARD CARE AND ADDITIONAL TO STANDARD CARE PROCEDURES

The only difference from "standard care" is in selecting the site of placement of temperature probe at the time of birth as detailed above. There is no other variation from standard care.

6.4 RANDOMISATION

Stratified randomisation with permuted blocks for assignment to groups within each stratum will be conducted to maintain balance and reduce sampling errors. The randomisation will be stratified in 2 gestation bands (23-27 weeks, 28-31 weeks). An opaque envelope will be used to conceal the group allocation and this will be opened in the birth unit/operating theatre just before the birth of the infant. The staff attending the delivery will open the envelope and assign the infant to one of the 2 groups. As the primary outcome is objectively collected soon after birth, there is no blinding of the investigators. For practical reasons, the clinical staff performing the resuscitation cannot be blinded.

6.5 STUDY METHODOLOGY

The study data will be collected from a review of the medical records of the mother and the infant. The data proposed to be collected are given below:

Maternal details:

History of maternal fever or hypothermia during labour with temperature

Risk factors for infection (such as positive GBS result, preterm rupture of membranes > 18hrs, clinical chorioamnionitis):

Receipt of antenatal steroids

Mode of delivery: Normal vaginal birth/vaginal with instruments/vaginal breech/LSCS

Maternal anaesthesia (Y//N) and spinal/ general

Maternal antibiotics (Y/N)

Neonatal details:

Gestational age:

Birth weight

Small for gestational age (weight <10th centile) (Y/N)

Singleton/multiple births

Gender

Apgar score at 1 min and 5 minutes

Resuscitation at birth:

- Suction/stimulation only
- Continuous positive airway pressure (CPAP)
- Intubation
- Chest compressions

Umbilical arterial cord gas

Environmental Temperature at birth

Time to NICU admission in minutes

Axillary temperature on admission (Primary outcome related)

Secondary outcomes:

Death

Major intra-ventricular haemorrhage (grade 3 or 4 IVH)

Early onset culture proven sepsis

Late onset culture proven sepsis

Stage II and above necrotising enterocolitis

Cystic peri-ventricular leucomalacia

Retinopathy of prematurity needing surgery

Neonatal chronic lung disease

7. Study Population

7.1 Recruitment Procedure

The study group includes all the preterm infants born at Westmead Hospital with a gestational age of 23^{+0} weeks to 31^{+6} weeks. The neonatal staff members are routinely required to attend these births. Medical and nursing staff will be informed about this study after the ethics approval. Envelopes for randomisation will be kept in an easily accessible area of the unit.

7.2 Inclusion Criteria

Preterm infants between 23^{+0} and 31^{+6} weeks gestation born at Westmead Hospital

7.3 Exclusion Criteria

Severe congenital malformations, severe asphyxia requiring cardiac compressions and adrenaline administration, birth prior to arrival of the NICU team, congenital cutaneous lesions

7.4 Consent

1. This is a minimal risk intervention study where placement of the temperature probe in the left armpit (standard of care) is being compared to placing the probe on the left upper back. This does not include any invasive procedure, treatment or invasive monitoring.
2. Privacy of the participants will be protected as per Ethics committee guidelines.
3. Data will be stored in password protected WSLHD computers.
4. Governance process as per current guidelines will be followed.
5. As participants are babies, parents will be consenting for their participation.

8. Participant Safety and Withdrawal

8.1 Risk Management and Safety

As the proposed intervention leads to a minimal alteration in care (choosing a different body site for probe placement for 20-30 minutes), we do not foresee any additional risk to the participants.

8.2 Adverse Event Reporting

The research team will perform regular audits of the admission temperatures of the preterm infants.

8.3 Handling of Withdrawals

NA

8.4 Replacements

NA

9. Statistical Methods

9.1 Sample Size Estimation & Justification

The data from our unit for 2016 shows that the proportion of infants <32 weeks gestation with normal admission temperature is 55%. If the experimental site of probe placement increases this proportion of infants to 75% a change in practice would be considered clinically worthwhile. A study of 178 babies (89 per group) will be able to detect this difference with 80% power assuming two-sided 5% alpha using a chi-square test.

9.2 Power Calculations

As above

9.3 Statistical Methods To Be Undertaken

Demographic and clinical characteristic and study outcomes will be described by randomised treatment and overall using standard statistical methods: frequencies and percentages for categorical variables and mean (standard deviation) or median (interquartile range) and range for continuous variables. The analysis will be by intention to treat principle. The primary outcome (proportion of infants with temperature in the normal range i.e. 36.8-37.3◦C) and secondary outcomes will be compared between the two groups using a chi-square test or appropriate exact test if required for rare outcomes. Differences will be described with appropriate confidence intervals where possible. Exploratory regression analyses may be used to examine predictors of outcome or to perform adjusted analyses.

Where assumptions of normality are not met, non-parametric alternatives will be undertaken.

10. Storage of Blood and Tissue Samples

10.1 Details of where samples will be stored, and the type of consent for future use of samples

NA

11. Data Security & Handling

11.1 Details of where records will be kept & How long will they be stored

The data will be stored in a WSLHD computer which will be password protected. The password will only be known to the members of the research team. The data file will be transferred among the research team members via secure email (only with the health email address). After the required period of storage (5 years), this data will be deleted.

11.2 Confidentiality and Security

The data will be stored in a password protected work computer and the password will be known only to the members of the research team.

11.3 Ancillary data

NA

12. Appendix

NA

List of Attachments included:

**Document Name**	**Version Number**	**Date (e.g., 18 January 2012)**
WSYD-PROC202201. Thermal Management of a Neonate born at or less than 32 weeks gestation and/or less than 1500gms	0.2	18 Jun 2015

13. References

1. Laptook AR, Salhab W, Bhaskar B; Neonatal Research N: Admission temperature of low birth weight infants: predictors and associated morbidities. Pediatrics 2007; 119:e643–e649.
2. Lyu Y, Shah PS, Ye XY, Warre R, Piedboeuf B, Deshpandey A, Dunn M, Lee SK; Canadian Neonatal Network: Association between admission temperature and mortality and major morbidity in preterm infants born at fewer than 33 weeks' gestation. JAMA Pediatr 2015; 169:e150277
3. Bensouda B, Mandel R, Mejri A, Lachapelle J, St-Hilaire M, Ali N. Temperature probe placement during preterm infants resuscitation: A randomised trial. Neonatology 2018;113:27-32

SuperScript

writing & editing

0425 381 984
olivia@superscriptwriting.com.au

27-Jun-23

I have given permission for my business to be named in the Acknowledgments section for editing this article.

Olivia Wroth, Principal
SuperScript Writing & Editing
